# Prescribed Performance Control of a Human-Following Surveillance Robot with Incomplete Observation

**DOI:** 10.34133/cbsystems.0085

**Published:** 2024-01-25

**Authors:** Meiping Zhang, Zixin Chi, Zhaohui Yang, Samer Mohammed, Jian Huang

**Affiliations:** ^1^ Department of Artificial Intelligence and Automation, Huazhong University of Science and Technology, Wuhan, China.; ^2^ Department of Rehabilitation Medicine, Union Hospital, Tongji Medical College, Huazhong University of Science and Technology, Wuhan, China.; ^3^ Laboratory of Image, Signal and Intelligent Systems, Univ Paris Est Creteil, LISSI, F-94400 Vitry, France.

## Abstract

For people with lower limb muscle weakness, effective and timely rehabilitation intervention is essential for assisting in daily walking and facilitating recovery. Numerous studies have been conducted on rehabilitation robots; however, some critical issues in the field of human-following remain unaddressed. These include potential challenges related to the loss of sensory signals for intention recognition and the complexities associated with maintaining the relative pose of robots during the following process. A human-following surveillance robot is introduced as the basis of the research. To address potential interruptions in motion signals, such as data transmission blockages or body occlusion, we propose a human walking intention estimation algorithm based on set-membership filtering with incomplete observation. To ensure uninterrupted user walking and maintain an effective aid and detection range, we propose a human-following control algorithm based on prescribed performance. The experiment verifies the effectiveness of the proposed methods. The proposed intention estimation algorithm achieves continuous and accurate intention recognition under incomplete observation. The control algorithm presented in this paper achieves constrained robot following with respect to the relative pose.

## Introduction

The World Health Organization points out that the increase in the global elderly population may lead to an increase in the number of patients with lower limb weakness. It is estimated that around 400 million older people worldwide suffer from muscle loss [1]. In addition to the natural decline of muscle strength in the elderly, lower limb muscle strength loss can also be caused by neurological diseases, such as spinal cord injury, and motor system diseases, such as degenerative joint disease.

Timely and accurate rehabilitation medical interventions can accelerate patient recovery, prevent disability or reduce its severity, enhance patients’ quality of life, and increase the likelihood of their successful reintegration into society. According to clinical medical data [2], 70% to 80% of people with lower limb muscle loss can recover some or even most of their motor functions after undergoing early treatment and rehabilitation training with timely and effective rehabilitation means. To meet the needs of patient groups and reduce the burden on medical institutions and doctors, related rehabilitation robots have increasingly become a research focus [3].

Prior studies have developed crutch robots designed to offer uninterrupted support to patients [4,5], specifically focusing on individuals with moderate muscle loss in their lower limbs and limited capacity for autonomous walking. These robots facilitate auxiliary motion by responding to the force signals generated when the user applies pressure to the handrails. This paper focuses on patients in the early stages of lower limb muscle strength decline, wherein they can generally walk independently but require monitoring to prevent deterioration resulting from potential falls. A rehabilitation robot developed for people with mild lower extremity muscle strength decline typically consists of easy-to-move parts, a human–robot interaction interface for capturing human motion intention, a support structure, a handrail, and both hardware and software systems [6]. The primary aim of this study is to design a robotic system that can autonomously track users with mild deterioration in lower limb muscle strength and provide handrail support as needed for balance. Beyond addressing lower limb muscle weakness, these mobile human-following robots hold substantial potential across various applications, including logistics and warehousing, domestic assistance, tourism, and recreational activities. The research presented in this paper aims to enhance the performance of human-following robots, potentially enabling them to better meet the needs of various uses.

Several lower limb rehabilitation robots have been developed to assist individuals with mild lower limb muscle weakness. These include the intelligent cane robot system SmartCane [7] proposed by Dubowsky et al. from Massachusetts Institute of Technology, the 2-wheeled cane robot RoJi [8] developed by Shoval and Shim et al., the walking aid robot ISR-AIWALKER [9] developed by the University of Coimbra in Portugal, and the omnidirectional cane robot iCane [10] developed by Nagoya University in Japan. However, several aspects have not been addressed in these studies. Firstly, it is essential to address the issue of maintaining the continuity and accuracy of human motion intention when the signals are temporarily disrupted during the acquisition of human motion data through the human–robot interaction interface. These circumstances may occur as a result of signal interference, often manifesting as data transmission blockages. Additionally, instances of signal interruption may occur during the scanning of human leg data using a laser rangefinder, such as when there is occlusion induced by leg crossing. The utilization of visual sensors for human body joint acquisition may also lead to this issue, such as when human body information is lost due to the change of light or the instability within the joint detection algorithm. Secondly, the development of an effective robot motion control strategy is crucial to maintain the robot’s close proximity to the individual for timely support, while ensuring that the robot does not cause any hindrance to the person during walking. Lastly, it is imperative to find ways to overcome the limitations of the sensor’s detection range so that the robot does not lose track of the person. These considerations are crucial for the improvement of robots designed to assist individuals with lower limb muscle weakness.

In this paper, the human walking intention recognition algorithm is designed by using the set-membership filter with incomplete observation [11]. This approach enables accurate recognition of the intention while addressing potential adverse effects due to temporary absence of walking signals. Furthermore, this paper proposes a human-following control method incorporating prescribed performance. Utilizing the convergence characteristics of the performance envelope function [12–15], this control method effectively governs both the transient and steady-state performance of the controlled system. As a result, the robot remains within the predefined constraint range, ensuring the delivery of safe and comfortable services to users while continuously detecting the user within the robot’s operational range. Only when safe and comfortable following is achieved can further research into user status monitoring and rehabilitation training be conducted.

The main contributions of this paper are summarized as follows: Firstly, a human walking intention estimation algorithm is introduced, utilizing set-membership filtering with incomplete observation. Secondly, a robot human-following control algorithm is proposed, incorporating prescribed performance. Lastly, the efficacy of the proposed approaches is verified through experimentation.

The subsequent sections of this paper are structured as follows: In Methods, the human-following surveillance robot platform is introduced, serving as the foundation for the conducted research, and the control method to achieve the robot’s design objectives is overviewed. Human Walking Intention Estimation details the novel human walking intention estimation algorithm, which relies on set-membership filtering while considering incomplete observation. Prescribed Performance Follow Control is dedicated to the analysis and design of the robot human-following control algorithm incorporating prescribed performance. Subsequently, Results validates the efficacy of the proposed methodology through experiments. Finally, Discussion succinctly provides a conclusion and outlines future works.

## Methods

### Structure of a human-following surveillance robot

**Fig. 1.**
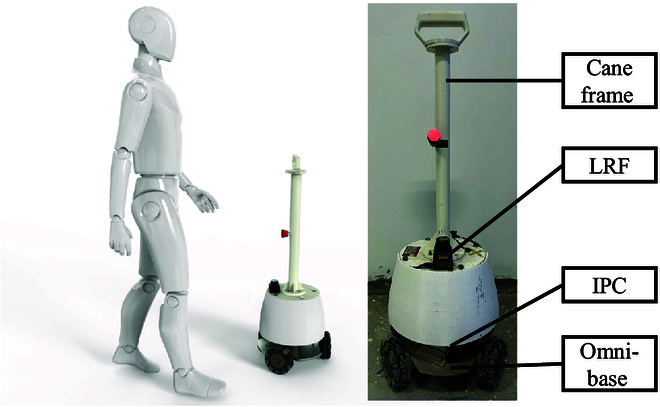
Human-following surveillance robot.

Figure 1 illustrates the key components of the human-following surveillance robot, which primarily consists of a metal cane frame, a laser rangefinder (HOKUYO UBG-04LX-F01), an industrial personal computer, and an omni-directional base. The omni-directional base consists of 3 omni-directional wheels, each of which is equipped with a direct current motor. The laser rangefinder has a scanning angle range of ±120° and a maximum scanning distance of 5,600 mm, which serves the purpose of collecting data related to human leg motion.

### Definition of a coordinate system

**Fig. 2.**
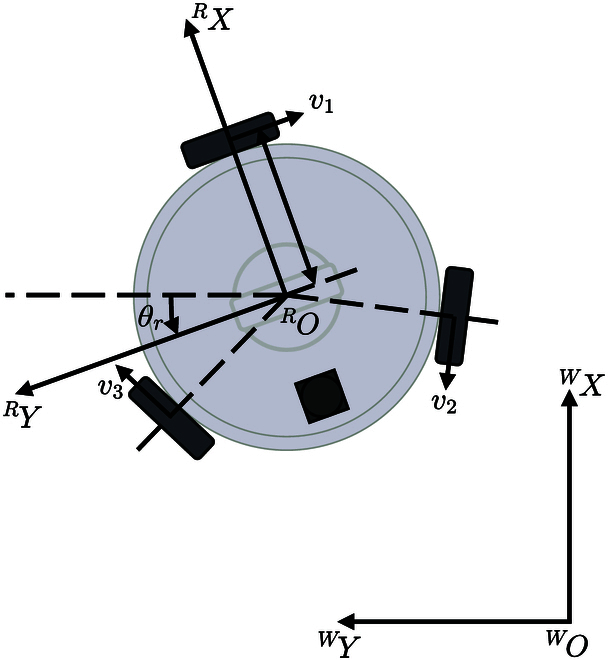
Schematic diagram of the coordinate system of the human-following surveillance robot.

The omni-directional mobile chassis structure of the human-following surveillance robot is depicted in Fig. 2. Establishing the robot coordinate system denoted as {*R*:*^R^O^R^X^R^Y^R^Z*}, the point *^R^O*, representing the projection of the center of the robot’s omni-directional mobile base on the ground, is chosen as the origin. In this coordinate system, the *^R^X* axis signifies the robot’s forward direction, while the *^R^Y* axis aligns with the horizontal leftward movement of the robot. Applying the right-hand rule, the direction of the *^R^Z* axis is determined as vertically upward. Simultaneously, the world coordinate system {*W*:*^W^O^W^X^W^Y^W^Z*} coincides with the robot coordinate system during the initial moment. As the robot moves, the position of the origin *^R^O* in the world coordinate system can be determined based on the robot’s pose in the world coordinate system, defined as $q_{r}={\left[x_{r}y_{r}\theta_{r}\right]}^{T}$. Herein, $x_{r}$ and $y_{r}$ denote the robot’s position in the world coordinate system, and $\theta_{r}$ represents the robot’s forward orientation in the world coordinate system.

Within the world coordinate system, the pose of the human body is denoted as $q_{h}={\left[x_{h}\;y_{h}\;\theta_{h}\right]}^{T}$, where $x_{h}$ and $y_{h}$ represent the human body’s positions, and $\theta_{h}$ represents the forward orientation.

### Kinematic model

The translational and rotational velocities of the omni-directional mobile base in the world coordinate system are derived by synthesizing the motion vectors of the 3 omni-directional continuous switching wheels. Hence, utilizing the mapping relationship between the rotational speed of each omni-directional continuous switching wheel and the pose of the robot’s center point, it becomes possible to establish the kinematics model of the omni-directional mobile base.

To elaborate further, let $L_{0}$ represent the vertical distance from the center of the omni wheel to the center of the robot, and let *r* denote the radius of the omni-directional wheel. The 3 omni-directional wheels within the omni-directional mobile base are designated in counterclockwise order, and the attitude of these wheels is defined as $\varphi ={\left[\varphi_{1}\;\varphi_{2}\;\varphi_{3}\right]}^{T}$, with angular velocity denoted as $\overset{\cdot }{\varphi }={\left[{\overset{\cdot }{\varphi }}_{1}\;{\overset{\cdot }{\varphi }}_{2}\;{\overset{\cdot }{\varphi }}_{3}\right]}^{T}$ and linear velocity denoted as ${}^{R}v={\left[v_{1}\;v_{2}\;v_{3}\right]}^{T}$. As a result, the velocity of the robot’s motion in the robot coordinate system {*R*} is denoted as ${}^{R}v_{r}={\left[{}^{R}v_{\mathrm{rx}}\;{}^{R}v_{\mathrm{ry}}\;{}^{R}\omega_{r}\right]}^{T}$.

According to the principle of motion vector synthesis, the relationship between the wheel linear velocity ${}^{R}v$ and the rotation angular velocity $\overset{\cdot }{\varphi }$ is as follows:

$${}^{R}v=r\overset{\cdot }{\varphi }$$ (1)

The relationship between the omnidirectional wheel linear velocity ${}^{R}v$ and the robot movement velocity ${}^{R}v_{r}$ is as follows:

$${}^{R}v={J_{r}}^{R}v_{r}$$ (2)

where $J_{r}=\left[\begin{array}{c} -\text{sin}\left(0\right)\\ -\text{sin}\left(\frac{2\pi }{3}\right)\\ -\text{sin}\left(\frac{4\pi }{3}\right) \end{array}\;\begin{array}{c} -\text{cos}\left(0\right)\\ -\text{cos}\left(\frac{2\pi }{3}\right)\\ -\text{cos}\left(\frac{4\pi }{3}\right) \end{array}\;\begin{array}{c} -L_{0}\\ -L_{0}\\ -L_{0} \end{array}\right]$. By incorporating Eqs. 1 and 2, the kinematics model of the robot can be expressed as follows:

$$\overset{\cdot }{\varphi }=\frac{1}{r}{J_{r}}^{R}v_{r}$$ (3)

### Design goals of the human-following surveillance robot

The objective of the human-following surveillance robot in this paper is to consistently track and accompany the user, ensuring prompt access to the robot’s support by enabling the user to grasp the cane when assistance is needed.

**Fig. 3.**
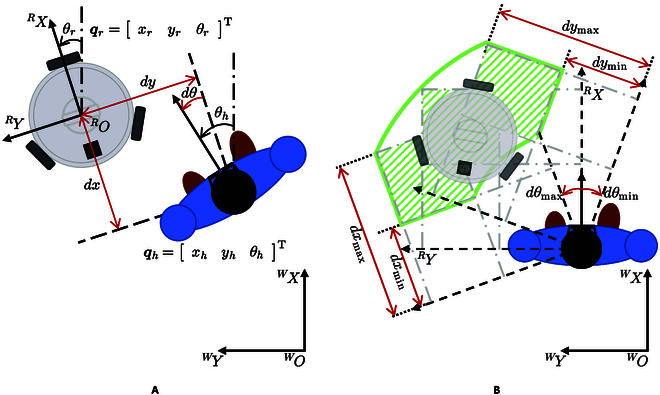
Schematic diagram of the robot following a human target. (A) Relative distance and orientation. (B) Robot’s prescribed performance.

As depicted in Fig. 3A, the relative distance between the robot and the user is defined as *dx* and *dy*. Additionally, the azimuthal angle *dθ* denotes the relative orientation between the robot and the user. It is straightforward to obtain the following.

$$\left[\begin{array}{c} \mathrm{dx}\\ \mathrm{dy}\\ d\theta \end{array}\right]=\left[\begin{array}{c} \text{cos}\theta_{r}\\ -\text{sin}\theta_{r}\\ 0 \end{array}\;\begin{array}{c} \text{sin}\theta_{r}\\ \text{cos}\theta_{r}\\ 0 \end{array}\;\begin{array}{c} 0\\ 0\\ 1 \end{array}\right]\left[\begin{array}{c} x_{h}-x_{r}\\ y_{h}-y_{r}\\ \theta_{h}-\theta_{r} \end{array}\right]$$ (4)

In accordance with the guidelines for traditional cane usage [16] and recommendations from clinicians, it is necessary for the robot to maintain a specific distance ${\mathrm{dx}}_{\mathrm{des}}$ from the user in the *^R^X* direction, ensuring that the robot remains within the reachable forward arm span of the user. Furthermore, considering the recommended use of the traditional cane, the robot should maintain a relatively fixed distance ${\mathrm{dy}}_{\mathrm{des}}$ from the user’s healthy side in the *^R^Y* direction. To facilitate timely user interaction with the robot’s handrail and ensure continuous user visibility, it is considered favorable and appropriate for the robot’s forward orientation to align with the forward orientation of the human body, indicating that the relative angle between the robot’s forward orientation and that of the human body, denoted as *dθ*_des_, should be maintained at *dθ*_des_ = 0^∘^.

However, due to transient and steady-state errors in the control system, maintaining an entirely fixed relative pose with the user at all times becomes challenging. Nevertheless, throughout the user’s walking process, the robot must maintain a balance, staying at an appropriate proximity to the user to prevent delays in cane grasping when needed, while avoiding being excessively close, as that could impede the user’s walking. Additionally, the robot must ensure that the user remains within its detection range. As a result, this paper proposes the following constraints:

$${\mathrm{dx}}_{\min}<\mathrm{dx}<{\mathrm{dx}}_{\max},\;{\mathrm{dy}}_{\min}<\mathrm{dy}<{\mathrm{dy}}_{\max},\;d\theta_{\min}<d\theta <{d\theta }_{\max}$$ (5)

where *dx*_min_ < *dx*_des_ < *dx*_max_ < 0, *dθ*_min_ < *dθ*_des_ < *dθ*_max_. If the user’s left leg is impaired, then 0 < *dy*_min_ < *dy*_des_ < *dy*_max_. Similarly, if the user’s right leg is impaired, then *dy*_min_ < *dy*_des_ < *dy*_max_ < 0. From the user’s perspective, the constraint range of the robot is shown in Fig. 3B, which can meet the use requirements of the robot.

### Overview of the control of the robot following the user

**Fig. 4.**
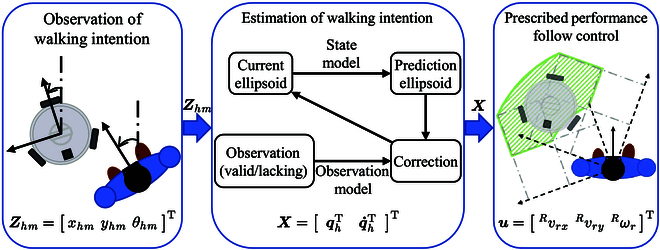
Overview of the control method.

An overview of the control method proposed to achieve the robot’s design objectives is shown in Fig. 4. The process of the robot following a person consists of the following 2 steps:

1. Estimation of human walking intention through the application of the set-membership filtering approach, while accounting for incomplete observation.

2. Implementation of prescribed performance control for the robot’s following function.

First, the human body pose ***Z****_hm_* = [*x_hm_ y_hm_ θ_hm_*]^T^ is measured through a single laser range finder. Subsequently, by using the measured data in conjunction with the intention estimation algorithm, the human walking intention $X={\left[q_{h}^{T}\;{\overset{\cdot }{q}}_{h}^{T}\right]}^{T}$ is derived. Finally, prescribed performance control is implemented, using the estimated intention to provide users with secure and comfortable following services.

## Human Walking Intention Estimation

To achieve the human-following function, the quantified human walking intention plays a central role as it determines the expected position and orientation of the human-following surveillance robot. However, potential information loss caused by body occlusion, data transmission loss, and other factors may lead to missing or inaccurate quantification of human walking intention. To address this, we propose a human walking intention estimation algorithm based on set-membership filtering with incomplete observation.

### Definition and state model of human walking intention

Accurate recognition of human walking intention is essential for effective robot motion control. Hence, we begin by giving a comprehensive definition of human walking intention. Human walking intention is characterized by a motion state vector, which includes the target user’s position, forward orientation, and speed:

$$X={\left[q_{h}^{T}\;{\overset{\cdot }{q}}_{h}^{T}\right]}^{T}$$ (6)

where $q_{h}={\left[x_{h}\;y_{h}\;\theta_{h}\right]}^{T}$ and ${\overset{\cdot }{q}}_{h}={\left[{\overset{\cdot }{x}}_{h}\;{\overset{\cdot }{y}}_{h}\;{\overset{\cdot }{\theta }}_{h}\right]}^{T}$ show the pose and velocity of the human body in the world coordinate system, respectively.

To effectively quantify and describe the evolution mechanism of intention across various walking modes, we present a unified evolution model of human walking intention. Within each very short sampling period Δ*T*, the human walking action can be treated as a uniform motion state. Based on the definition of human walking intention (Eq. 6), the state model of human walking intention can be expressed as follows:

$$X\left(k+1\right)=A\left(k\right)X\left(k\right)+w\left(k\right)$$ (7)

where $A\left(k\right)=\left[\begin{array}{c} I_{3}\\ 0 \end{array}\;\begin{array}{c} \Delta TI_{3}\\ I_{3} \end{array}\right]$. ***I***_3_ represents a 3 × 3 unit matrix. ***w***(*k*) denotes the process noise, which is bounded and belongs to the set Ω(0, ***Q***, *σ_w_*).

### Observation model of human walking intention

The observation of human walking intention is defined as:

$$Z_{\mathrm{hm}}={\left[x_{\mathrm{hm}}\;y_{\mathrm{hm}}\;\theta_{\mathrm{hm}}\right]}^{T}$$ (8)

where *x_hm_*, *y_hm_*, and *θ_hm_* represent the online obtained human body pose observation data through the laser rangefinder.

For the detection of the user’s position and attitude, a single laser rangefinder is installed in the negative direction of the *^R^X* axis. The Neighbor Distance Support Vector Domain Description method is utilized to extract information about the user’s 2 legs. The position of the human body $\left(x_{\mathrm{hm}},y_{\mathrm{hm}}\right)$ is calculated as the midpoint of the line connecting the legs. Additionally, the forward orientation of the human body, denoted as $\theta_{\mathrm{hm}}$, is determined based on either the vertical direction of the line connecting the legs or the speed direction of the midpoint of the line connecting the legs. This orientation varies in accordance with different walking modes [17].

Nevertheless, while the robot follows the user’s healthy limb side, the user’s legs may cross back and forth during walking, leading to occasional blockage of the other limb. As a result, the observed variables of the human walking intention become unattainable in such scenarios, leading to incomplete observation. Therefore, the observation model of human walking intention is described as:

$$Z_{\mathrm{hm}}\left(k\right)=\lambda \left(k\right)\left(\mathrm{HX}\left(k\right)+\tau \left(k\right)\right)$$ (9)

where $H=\left[\begin{array}{ccc} 1 & 0 & 0\\ 0 & 1 & 0\\ 0 & 0 & 1 \end{array}\;\begin{array}{ccc} 0 & 0 & 0\\ 0 & 0 & 0\\ 0 & 0 & 0 \end{array}\right]$, which is the state transition matrix of the human walking intention observation model. ***τ***(*k*) represents the measurement noise, which is bounded and belongs to the set Ω(0, ***U***, *γ*). If *λ*(*k*) = 1, it indicates that ***Z****_hm_*(*k*) is available for measurement, whereas when *λ*(*k*) = 0, it indicates that ***Z****_hm_*(*k*) is missing or unavailable for observation.

### Estimation of human walking intention

As mentioned earlier, this paper utilizes information from the legs scanned by a single laser rangefinder to derive the observation variables related to the user’s walking intention. However, during this process, the observation results may be missing due to the occlusion caused by leg crossings. Conventional filtering methods are insufficient in addressing this issue. Hence, in this section, a set-membership filtering algorithm with incomplete observation is introduced to calculate human walking intention.

Set-membership filtering is a recursive state boundary method that involves updating the feasible set. This set contains all potential state values that adhere to both the state equation and the observation equation and is updated as successive observations are received. In this paper, an ellipsoid is used to approximate the feasible set. We choose, as most do, the center of the ellipsoid as the state of the present moment. The algorithm based on the outer boundary of the ellipsoid offers advantages in terms of simplicity and high computational efficiency [18–20].

An ellipsoid can be defined as a set:

$$\Omega =\left\{x:{\left(x-a\right)}^{T}P^{-1}\left(x-a\right)\leq \sigma^{2}\right\}$$ (10)

where *a* ∈ R*^n^* represents the center of the ellipsoid, *x* ∈ R*^n^* is any point within the ellipsoid, ***P*** ∈ R^*n*×*n*^ is a positive definite matrix that determines the shape of the ellipsoid, and *σ* ∈ R denotes a measure of the size of the ellipsoid. This ellipsoid is denoted as Ω(*a*, ***P***, *σ*) in the context of this paper. Notably, both process noise and observation noise are within the set of known ellipsoids.

First, at *t* = *k* + 1, the surrounding ellipsoid $\Omega \left(\hat{X}\left(k\right),P\left(k\right),\sigma \left(k\right)\right)$ of ***X***(*k*) at *t* = *k* is known.

According to the human walking intention state model (Eq. 7), the prediction ellipsoid Ω(*k* + 1|*k*) = Ω(***X***(*k* + 1|*k*), ***P***(*k* + 1|*k*), *σ*(*k* + 1|*k*)), which contains the state at *t* = *k* + 1, can be obtained, where

$$X\left(k+1\left|k\right.\right)=A\left(k\right)\hat{X}\left(k\right)$$ (11)

$$\sigma {\left(k+1\left|k\right.\right)}^{2}=\sigma {\left(k\right)}^{2}$$ (12)

$$\begin{array}{c} P\left(k+1\left|k\right.\right)=\left(1+p\left(k\right)\right)A\left(k\right)P\left(k\right)A{\left(k\right)}^{T}+\left(1+p{\left(k\right)}^{-1}\right)\frac{\sigma_{w}^{2}}{\sigma {\left(k+1\left|k\right.\right)}^{2}}Q \end{array}$$ (13)

$$p\left(k\right)=\frac{\sigma_{w}}{\sigma \left(k\right)}\sqrt{\frac{\mathrm{tr}\left(Q\right)}{\mathrm{tr}\left(A\left(k\right)P\left(k\right)A{\left(k\right)}^{T}\right)}}$$ (14)

***A***(*k*), ***Q***, and *σ_w_* are as described in Eq. 7.

Next, the prediction results are updated and corrected based on the observation outcomes. There are 2 possible cases based on the presence or absence of observation variables.

Case 1: When the observed variable is not missing, that is *λ*(*k*) = 1, the ellipsoid $\Omega \left(\hat{X}\left(k+1\right),P\left(k+1\right),\sigma \left(k+1\right)\right)$,representing the final estimated state at *t* = *k* + 1, can be obtained, where

$$P{\left(k+1\right)}^{-1}=P{\left(k+1\left|k\right.\right)}^{-1}+q\left(k+1\right)H^{T}H$$ (15)

$$\begin{array}{c} \sigma {\left(k+1\right)}^{2}=\sigma {\left(k+1\left|k\right.\right)}^{2}+q\left(k+1\right)\gamma^{2}\\ \;-q\left(k+1\right)e{\left(k+1\right)}^{T}S{\left(k+1\right)}^{-1}e\left(k+1\right) \end{array}$$ (16)

$$\hat{X}\left(k+1\right)=X\left(k+1\left|k\right.\right)+q\left(k+1\right)P\left(k+1\right)H^{T}e\left(k+1\right)$$ (17)

$$S\left(k+1\right)=U+q\left(k+1\right)\mathrm{HP}\left(k+1\left|k\right.\right)H^{T}$$ (18)

$$e\left(k+1\right)=Z_{\mathrm{hm}}\left(k\right)-\mathrm{HX}\left(k+1\left|k\right.\right)$$ (19)

$$\begin{array}{c} q\left(k+1\right)=\left\{\begin{array}{c} 0,\left|e\left(k+1\right)\right|\leq \gamma \\ \frac{1}{g\left(k+1\right)}\left(\frac{\left|e\left(k+1\right)\right|}{\gamma }-1\right),\left|e\left(k+1\right)\right|>\gamma \end{array}\right. \end{array}$$ (20)

***H***, ***U***, and *γ* are as described in Eq. 9. *g*(*k* + 1) is the largest singular value of ***HP***(*k* + 1|*k*)***H***^T^.

Case 2: When the observed variable is missing due to factors like body occlusion and data transmission loss, that is, *λ*(*k*) = 0, the prediction ellipsoid $\Omega \left(\hat{X}\left(k+1\right),\;P\left(k+1\right),\;\sigma \left(k+1\right)\right)$ is chosen as the ellipsoid representing the final estimated state at *t* = *k* + 1.

$$\begin{array}{c} P\left(k+1\right)=P\left(k+1\left|k\right.\right),\sigma {\left(k+1\right)}^{2}\\ \;=\sigma {\left(k+1\left|k\right.\right)}^{2},\hat{X}\left(k+1\right)=X\left(k+1\left|k\right.\right) \end{array}$$ (21)

Using the ellipsoid set-membership filter with incomplete observation, the estimation of human walking intention can be carried out by integrating the state model and the observation model. This approach enables the acquisition of a more accurate, stable, and seamless representation of walking intention, particularly in situations with missing data. The estimated intention serves as crucial input for subsequent follow control, contributing to improved performance and reliability in the robot’s following function.

## Prescribed Performance Follow Control

To facilitate the human-following surveillance robot’s following function, ensuring the user’s ability to access the cane at any time, preventing the user from experiencing obstruction by the robot, and avoiding the robot losing track of the user due to limited detection range, we introduce a prescribed performance follow control algorithm. This control algorithm imposes constraints on the maximum and minimum distances and azimuths between the robot and the user.

Initially, through the coordinate system transformation from {*R*} to {*W*}, the following relationships can be established:

$${\overset{\cdot }{q}}_{r}=J{\left(\theta_{r}\right)}^{R}v_{r}$$ (22)

where $J\left(\theta_{r}\right)=\left[\begin{array}{ccc} \text{cos}\left(\theta_{r}\right) & -\text{sin}\left(\theta_{r}\right) & 0\\ \text{sin}\left(\theta_{r}\right) & \text{cos}\left(\theta_{r}\right) & 0\\ 0 & 0 & 1 \end{array}\right].$

Based on the estimated human walking intention $X={\left[q_{h}^{T}\;{\overset{\cdot }{q}}_{h}^{T}\right]}^{T}$, the corresponding estimated posture and velocity can be obtained as ***q****_h_* = [*x_h_  y_h_  θ_h_*]^T^ and ${\overset{\cdot }{q}}_{h}={\left[{\overset{\cdot }{x}}_{h}\;{\overset{\cdot }{y}}_{h}\;{\overset{\cdot }{\theta }}_{h}\right]}^{T}$, respectively.

Define the relative distance and angle errors as follows:

$$e_{\mathrm{dx}}\left(t\right)=\mathrm{dx}\left(t\right)-{\mathrm{dx}}_{\mathrm{des}}$$ (23)

$$e_{\mathrm{dy}}\left(t\right)=\mathrm{dy}\left(t\right)-{\mathrm{dy}}_{\mathrm{des}}$$ (24)

$$e_{\mathrm{d\theta}}\left(t\right)=\mathrm{d\theta}\left(t\right)-{d\theta }_{\mathrm{des}}$$ (25)

In accordance with the formulated overall objective for the robot, achieving compliance with the relative distance constraint and relative azimuth constraint (Eq. 5) requires the satisfaction of the following inequalities:

$${\mathrm{dx}}_{\min}-{\mathrm{dx}}_{\mathrm{des}}<e_{\mathrm{dx}}\left(t\right)<{\mathrm{dx}}_{\max}-{\mathrm{dx}}_{\mathrm{des}},\forall t\geq 0$$ (26)

$${\mathrm{dy}}_{\min}-{\mathrm{dy}}_{\mathrm{des}}<e_{\mathrm{dy}}\left(t\right)<{\mathrm{dy}}_{\max}-{\mathrm{dy}}_{\mathrm{des}},\forall t\geq 0$$ (27)

$$d\theta_{\min}-d\theta_{\mathrm{des}}<e_{\mathrm{d\theta}}\left(t\right)<d\theta_{\max}-d\theta_{\mathrm{des}},\forall t\geq 0$$ (28)

Furthermore, to limit the bounds of inequalities (Eqs. 26 to 28), the relative distance and angle errors are subject to the following time-varying constraints:

$$\begin{array}{l} -\rho_{\mathrm{dj}}\left(t\right)<e_{\mathrm{dj}}\left(t\right)<\rho_{\mathrm{dj}}\left(t\right),j=x,y,\theta \\ \rho_{\mathrm{dj}}\left(t\right)=\left(\rho_{\mathrm{dj},0}-\rho_{\mathrm{dj},\infty }\right)e^{-k_{\mathrm{dj}}t}+\rho_{\mathrm{dj},\infty } \end{array}$$ (29)

where *ρ*_*dj*,0_ > 0, *ρ*_*dj*,∞_ > 0, *k_dj_* > 0(*j* = *x*, *y*, *θ*). The boundary function *ρ_dj_*(*t*) is an exponential decay function of time, indicating that its maximum value is attained at the initial time. By adhering to these conditions *ρ*_*dj*,0_ =  min {*dj*_des_ − *dj*_min_, *dj*_max_ − *dj*_des_}, *j* = *x*, *y*, *θ*, it becomes possible to fulfill the prescribed constraints (Eqs. 26 to 28), while also ensuring the fulfillment of the specified performance requirements outlined in Eq. 5.

The derivations of Eqs. 23 to 25 and their subsequent combination with Eqs. 4 and 22 leads to the following results:

$$\begin{array}{c} {\overset{\cdot }{e}}_{\mathrm{dx}}={-}^{R}\;v_{\mathrm{rx}}+{\left(y_{h}\text{cos}\theta_{r}-y_{r}\text{cos}\theta_{r}-x_{h}\text{sin}\theta_{r}+x_{r}\text{sin}\theta_{r}\right)}^{R}\omega_{r}\\ \;+\left({\overset{\cdot }{x}}_{h}\text{cos}\theta_{r}+{\overset{\cdot }{y}}_{h}\text{sin}\theta_{r}\right) \end{array}$$ (30)

$$\begin{array}{c} {\overset{\cdot }{e}}_{\mathrm{dy}}={-}^{R}\;v_{\mathrm{ry}}+{\left(-y_{h}\text{sin}\theta_{r}+y_{r}\text{sin}\theta_{r}-x_{h}\text{cos}\theta_{r}+x_{r}\text{cos}\theta_{r}\right)}^{R}\omega_{r}\\ \;+\left(-{\overset{\cdot }{x}}_{h}\text{sin}\theta_{r}+{\overset{\cdot }{y}}_{h}\text{cos}\theta_{r}\right) \end{array}$$ (31)

$${\overset{\cdot }{e}}_{\mathrm{d\theta}}={-}^{R}\;\omega_{r}+{\overset{\cdot }{\theta }}_{h}$$ (32)

Consider a tangent barrier Lyapunov function:

$$V_{\mathrm{dj}}=\frac{\rho_{\mathrm{dj}}^{2}}{\pi }\text{tan}\frac{\pi e_{\mathrm{dj}}^{2}}{2\rho_{\mathrm{dj}}^{2}},j=x,y,\theta$$ (33)

The tangent barrier Lyapunov function exhibits a notable finite escape property, indicating that as the error $e_{\mathrm{dj}}$ approaches the specified error bounds $\rho_{\mathrm{dj}}$ and $-\;\rho_{\mathrm{dj}}$, the Lyapunov function *V* tends toward infinity. By ensuring that the barrier Lyapunov function remains bounded along the system trajectory [12], constraints (Eq. 29) are effectively met while simultaneously ensuring the desired transient and steady-state performance.

The derivation of Eq. 33 is as follows:

$$\begin{array}{c} {\overset{\cdot }{V}}_{\mathrm{dj}}=\left(1+{\text{tan}}^{2}\frac{\pi e_{\mathrm{dj}}^{2}}{2\rho_{\mathrm{dj}}^{2}}\right)e_{\mathrm{dj}}{\overset{\cdot }{e}}_{\mathrm{dj}}\\ \;+\frac{2\rho_{\mathrm{dj}}{\overset{\cdot }{\rho }}_{\mathrm{dj}}}{\pi }\text{tan}\frac{\pi e_{\mathrm{dj}}^{2}}{2\rho_{\mathrm{dj}}^{2}}-\frac{{\overset{\cdot }{\rho }}_{\mathrm{dj}}}{\rho_{\mathrm{dj}}}\left(1+{\text{tan}}^{2}\frac{\pi e_{\mathrm{dj}}^{2}}{2\rho_{\mathrm{dj}}^{2}}\right)e_{\mathrm{dj}}^{2},j=x,y,\theta \end{array}$$ (34)

Combined with Eqs. 30 to 32, take $\left({}^{R}v_{\mathrm{rx}},\;{}^{R}v_{\mathrm{ry}},\;{}^{R}\omega_{r}\right)$ as

$${}^{R}\omega_{r}={\overset{\cdot }{\theta }}_{h}-C_{d\theta }$$ (35)

$$\begin{array}{c} {}^{R}v_{\mathrm{rx}}={\left(y_{h}\text{cos}\theta_{r}-y_{r}\text{cos}\theta_{r}-x_{h}\text{sin}\theta_{r}+x_{r}\text{sin}\theta_{r}\right)}^{R}\omega_{r}\\ \;+\left({\overset{\cdot }{x}}_{h}\text{cos}\theta_{r}+{\overset{\cdot }{y}}_{h}\text{sin}\theta_{r}\right)-C_{\mathrm{dx}} \end{array}$$ (36)

$$\begin{array}{c} {}^{R}v_{\mathrm{ry}}={\left(-y_{h}\text{sin}\theta_{r}+y_{r}\text{sin}\theta_{r}-x_{h}\text{cos}\theta_{r}+x_{r}\text{cos}\theta_{r}\right)}^{R}\omega_{r}\\ \;+\left(-{\overset{\cdot }{x}}_{h}\text{sin}\theta_{r}+{\overset{\cdot }{y}}_{h}\text{cos}\theta_{r}\right)-C_{\mathrm{dy}} \end{array}$$ (37)

where $\begin{array}{c} C_{\mathrm{dj}}=b_{\mathrm{dj}}\frac{\rho_{\mathrm{dj}}^{2}}{\pi e_{\mathrm{dj}}}\text{sin}\frac{\pi e_{\mathrm{dj}}^{2}}{2\rho_{\mathrm{dj}}^{2}}\text{cos}\frac{\pi e_{\mathrm{dj}}^{2}}{2\rho_{\mathrm{dj}}^{2}}+b_{\mathrm{dj}1}e_{\mathrm{dj}},j=x,y,\theta \end{array}$. *b_dj_* and *b*_*dj*1_ are normal constants and $b_{\mathrm{dj}}>2b_{\mathrm{dj}1},b_{\mathrm{dj}1}>\frac{\left(\rho_{\mathrm{dj},0}-\rho_{\mathrm{dj},\infty }\right)k_{\mathrm{dj}}}{\rho_{\mathrm{dj},\infty }}$.

Upon substituting Eqs. 35 to 37 into Eq. 34, the resulting expressions are as follows:

$$\begin{array}{c} \begin{array}{c} {\overset{\cdot }{V}}_{\mathrm{dj}}=\left(-b_{\mathrm{dj}}+\frac{2{\overset{\cdot }{\rho }}_{\mathrm{dj}}}{\rho_{\mathrm{dj}}}\right)V_{\mathrm{dj}}+\left(1+{\text{tan}}^{2}\frac{\pi e_{\mathrm{dj}}^{2}}{2\rho_{\mathrm{dj}}^{2}}\right)e_{\mathrm{dj}}^{2}\left(-b_{\mathrm{dj}1}-\frac{{\overset{\cdot }{\rho }}_{\mathrm{dj}}}{\rho_{\mathrm{dj}}}\right)\\ \leq -\left(b_{\mathrm{dj}}-2b_{\mathrm{dj}1}\right)V_{\mathrm{dj}},j=x,y,\theta \end{array} \end{array}$$ (38)

Since $V_{\mathrm{dj}}\geq 0$, ${\overset{\cdot }{V}}_{\mathrm{dj}}\leq 0$ is satisfied, the system can be asymptotically stable by the Lyapunov stability criterion. Finally, based on the robot kinematics model (Eq. 3), the control directives for the robot chassis can be obtained.

## Results

### Experimental setup

**Fig. 5.**
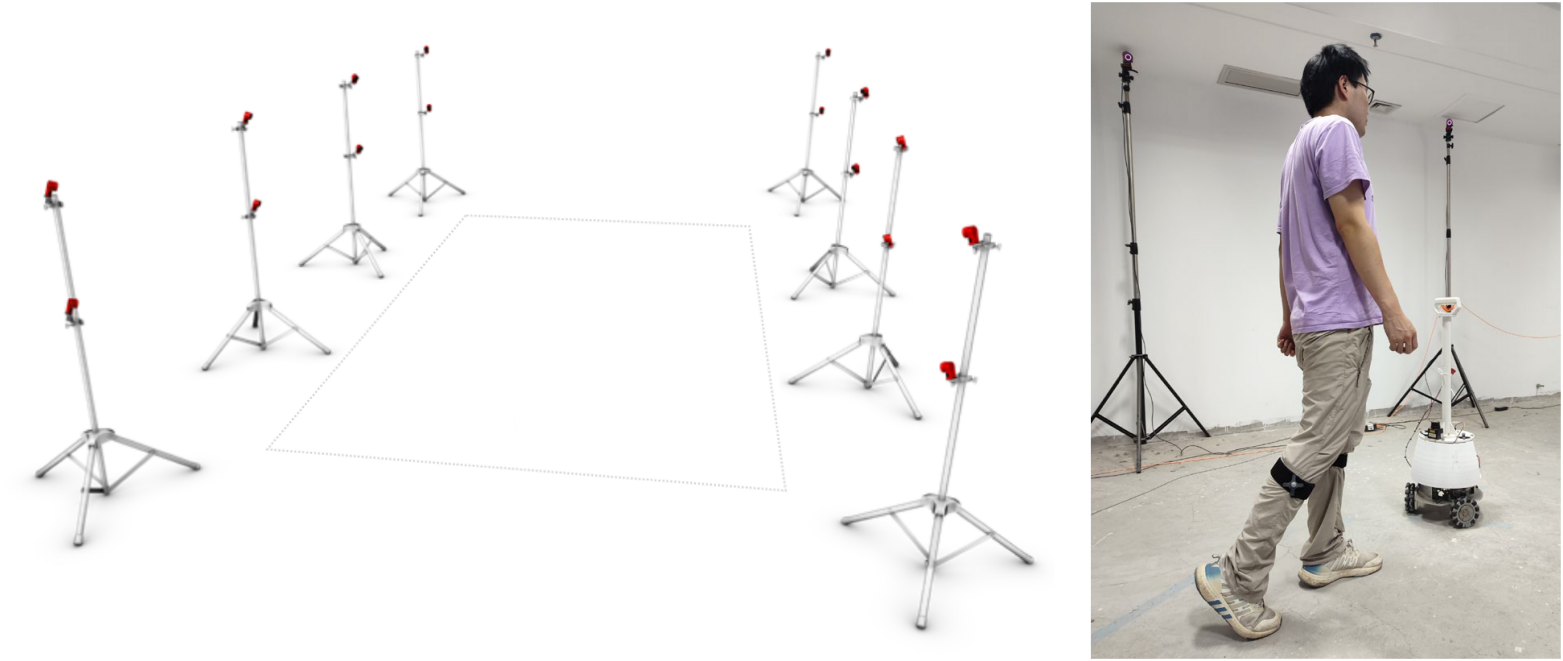
OptiTrack platform and experiments.

OptiTrack platform was used for conducting verification experiments, as illustrated in Fig. 5. OptiTrack serves as a vision-based motion capture system featuring smart cameras with processor boards and built-in high-precision marker processing algorithms. The high detection accuracy of OptiTrack allows the obtained motion data to closely approximate the actual motion data, making it suitable as a reference for assessing and validating human walking intention estimation. The OptiTrack system used in this experimental environment has 8 cameras, high resolution (1,280 × 1,024), and low latency (8.3 ms). The identified frame rates and 3D mean errors are 120 Hz and 0.75 mm, respectively.

It is crucial to maintain a reflective point-free environment within the OptiTrack platform since the cameras capture the positions and displacements of the reflective markers, that move in sync with the observed objects, enabling OptiTrack to record the trajectory of these markers during motion.

### Experimental results of human walking intention estimation

The verification experiment of walking intention estimation in the human-following process is conducted. Throughout the experiment, the robot utilizes a laser rangefinder to collect the motion signal of the human body and applies the proposed walking intention estimation method to determine the human intention. The parameters of the set-membership filter are set to $T=\frac{1}{10}s$, ***Q***(*k*) = *I*_6_, *σ_w_* = 0.2, and *γ* = 0.08. By comparing the detection data before filtering, the estimated data after filtering, and the real data collected by Optitrack during walking, the effectiveness of the proposed intention estimation method can be verified.

**Fig. 6.**
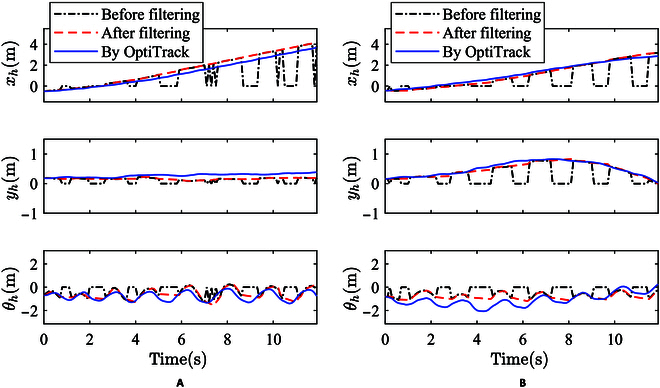
Walking intention estimation results. (A) Walking straight. (B) Turning.

As shown in Fig. 6, the pose estimation results of human walking straight and turning are respectively shown. From the detection data before filtering, it can be observed that there is a periodic loss of detection information, resulting from occlusions caused by leg crossings during walking, which the laser rangefinder scanning captures. In cases of missing data, zero values are inserted. The estimated data after filtering basically eliminates the influence caused by information loss, thus facilitating the continuous inference of ambulatory intent. Moreover, when compared with the real data collected by Optitrack, the error is relatively small and the estimation is accurate. In addition, it could be noted that due to the slight swing of the walking posture, the human orientation angle shows periodic oscillations.

The experimental results demonstrate that the intention estimation algorithm formulated in this paper effectively achieves the intended objective. It consistently and accurately recognizes walking intention, thereby validating its efficacy.

### Experimental results of prescribed performance follow control

The verification experiment of the robot’s prescribed performance following control is performed. The partial parameters of the prescribed performance following control are set as follows: *dx*_des_ =  −0.6, *dx*_max_ =  −0.3, *dx*_min_ =  −0.9, *dy*_des_ =  −0.6, *dy*_max_ = −0.3, *dy*_min_ =  −0.9, *dθ*_des_ = 0, $d\theta_{\max}=\frac{\pi }{6}$, and $d\theta_{\min}=-\;\frac{\pi }{6}$. The initial errors of the straight-walking experiment are as follows: *dx* =  −0.25, *dy* = 0.25, and $\mathrm{d\theta}=\frac{\pi }{8}$. The initial errors of the turn-walking experiment are as follows: *dx* =  −0.25, *dy* = 0.25, and $\mathrm{d\theta}=-\;\frac{\pi }{8}$. The effectiveness of the proposed control method is verified by evaluating whether the relative position and angle comply with the prescribed constraint range.

**Fig. 7.**
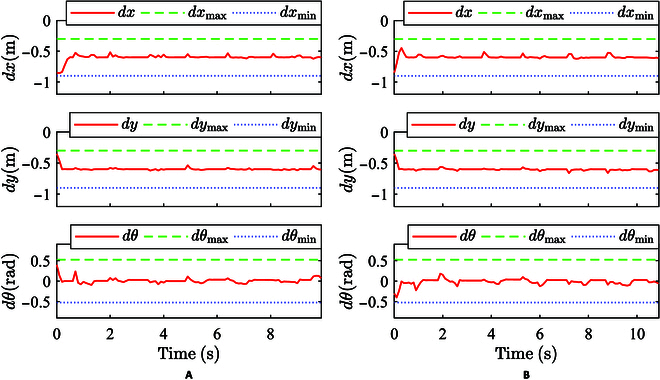
Follow control results. (A) Walking straight. (B) Turning.

**Fig. 8.**
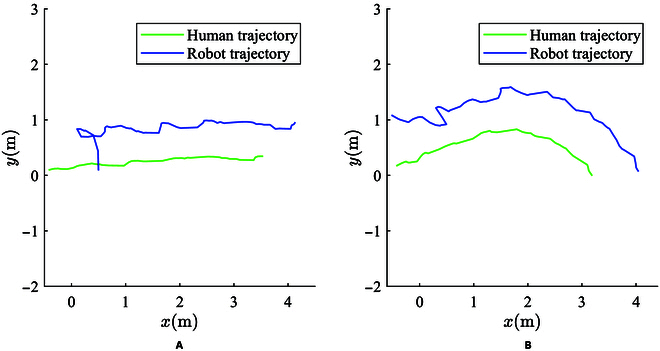
Follow control trajectory. (A) Walking straight. (B) Turning.

As shown in Fig. 7, the control results in the process of the robot following the user are shown. From the monitoring results of relative position and angle, it can be observed that the following trajectory of the robot complies well with the constraint range prescribed in the method and is basically stable at the ideal relative pose. At the beginning of the experiment, the relative pose of the user and the robot deviated from the ideal relative pose with a certain error, and quickly converged to the ideal relative pose after the experiment began, as can be also observed from the trajectory of the robot and the user in Fig. 8. It should be noted that certain segments of the robot’s trajectories exhibit less smoothness compared to those executed by humans, particularly in the initial phase of the experiment illustrated in Fig. 8B. This situation arises from the robot’s dual objectives: It should not only maintain a consistent distance from the human but also maintain a relatively constant orientation angle with the human. This strategic positioning ensures that the robot consistently maintains a forward position alongside the human, thereby delivering a comfortable level of assistance. When a person’s orientation deviates from the walking direction, in order to maintain a constant relative angle, the robot must make more substantial adjustments in its position and orientation, leading to a noticeable fluctuation in its trajectory.

The experimental results demonstrate that the prescribed performance following control algorithm designed in this paper achieves the expected goal and further fulfills the design goal of the human-following surveillance robot, thus confirming its effectiveness.

## Discussion

The lack of motion information in this study arises from the limitation that the laser rangefinder cannot obtain leg data due to leg crossings. In nearly half of the instances, valid data are unavailable, yet the intention estimation algorithm effectively tackles the challenges of incomplete observations. It shows that the algorithm can deal with the problem of intention recognition with incomplete observation. This type of problem is not uncommon in our other studies. For instance, our use of vision sensors to capture depth and color images of individuals, combined with joint detection algorithms to determine human joint positions for inferring walking intentions, may sometimes face data loss due to either the absence of depth information or instability in the joint detection algorithm. This algorithm can be explored as a potential solution to address these challenges.

Regarding robot human-following control, we introduce a prescribed performance approach to balance robot proximity and safety in relation to the user. Experimental results confirm the capability of this control method to consistently maintain an appropriate relative position and orientation when the robot follows the user, thus effectively meeting the user’s needs. Beyond its applicability in following and monitoring individuals with mild lower extremity weakness, this research has broad implications for the deployment of human-following robots in various collaborative and assistive roles. For instance, robots aiding industrial workers can enhance work efficiency and safety.

In conclusion, this paper introduces the development of a human-following surveillance robot for individuals with weakened lower limb muscle strength who can walk independently. The robot is designed to track the user while maintaining a fixed relative pose. To identify the user’s walking intention, the proposed method establishes both state and observation models of human walking. The ellipsoid set-membership filter algorithm with incomplete observation is used to cope with lack of data in estimating the human’s walking intention. To achieve the desired robot following function, performance constraints are imposed on the relative distance and angle, and control laws for the robot are designed based on Lyapunov stability theory. Experimental validation is conducted to demonstrate the effectiveness of the intention recognition and the prescribed performance control.

Furthermore, it is crucial to acknowledge the limitations of this study. All experiments were conducted with healthy subjects, and not with actual patients exhibiting mild lower limb muscle strength reduction. Additionally, the experimental settings were confined to controlled indoor environments. Future investigations should involve experiments in more complex and diverse scenarios. Further research will also be conducted on how to monitor the user’s status and perform rehabilitation training.

## Data Availability

The data underlying this article will be shared on reasonable request to the corresponding author.
